# Supplementary material for: Identification of novel bromodomain inhibitors of Trypanosoma cruzi bromodomain factor 2 (TcBDF2) using a fluorescence polarization-based high-throughput assay
Source: Antimicrob Agents Chemother. 2024 Jul 19;68(8):e00243-24. doi: 10.1128/aac.00243-24 (PMC11304739; doi:10.1128/aac.00243-24)
Supplement: Supplemental material — Figures S1 to S7. [file aac.00243-24-s0001.pdf]

**SUPPLEMENTARY METHODS AND FIGURES FOR : Identification of novel Bromodomain inhibitors of Trypanosoma cruzi Bromodomain Factor 2 (TcBDF2) using a fluorescence polarization-base high-throughput assay**

Luis E. Tavernelli,[1,3,†] Victoria L. Alonso,[1,2] Imanol Peña,[3] Elvio Rodríguez Araya,[1,2] Romina Manarin,[2] Juan Cantizani,[3] Julio Martin,[3,‡] Juan Salamanca,[3] Paul Bamborough,[4] Felix Calderón,[3] Raquel Gabarro,[3] Esteban Serra [1,2]

1 Instituto de Biología Molecular y Celular de Rosario, CONICET, Rosario, Argentina.

2 Facultad de Ciencias Bioquímicas y Farmacéuticas, Universidad Nacional de Rosario, Rosario, Argentina.

3 GlaxoSmithKline Global Health, Tres Cantos, 28760, Madrid, Spain.

4 Molecular Design, GlaxoSmithKline, Gunnels Wood Road, Stevenage, Herts, SG1 2NY, UK

† Current address: School of Infection and Immunity. University of Glasgow, Glasgow, Scotland, UK.

‡ Current address: Sciengement Lab Consulting, 28750 San Agustin del Guadalix, Spain.

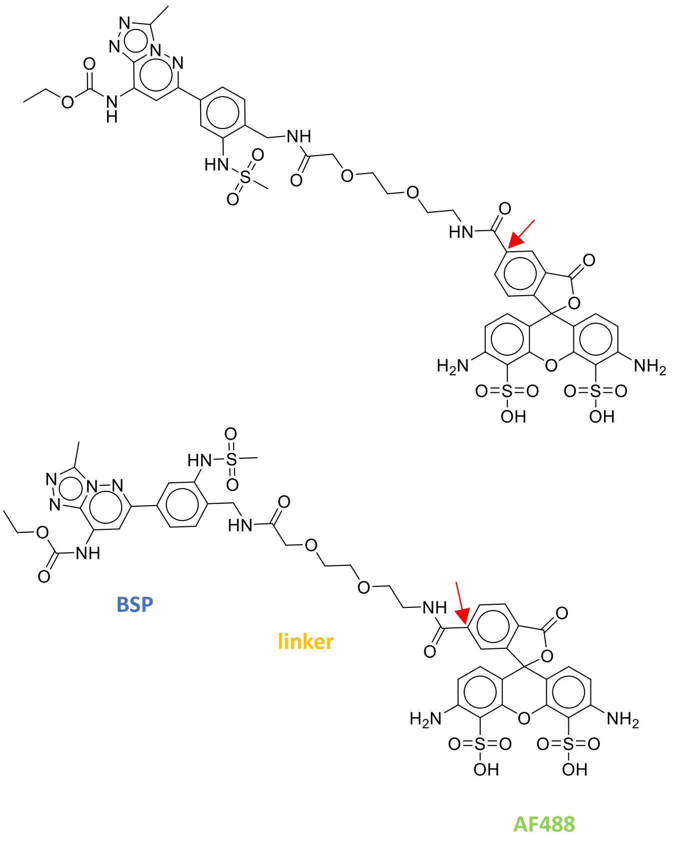


**Figure S1: AlexaFluor-488 coupled to BSP.** Mixture of regioisomers that differ at the atom on the benzene ring where the linker is bonded to the fluorophore (indicated with red arrows).

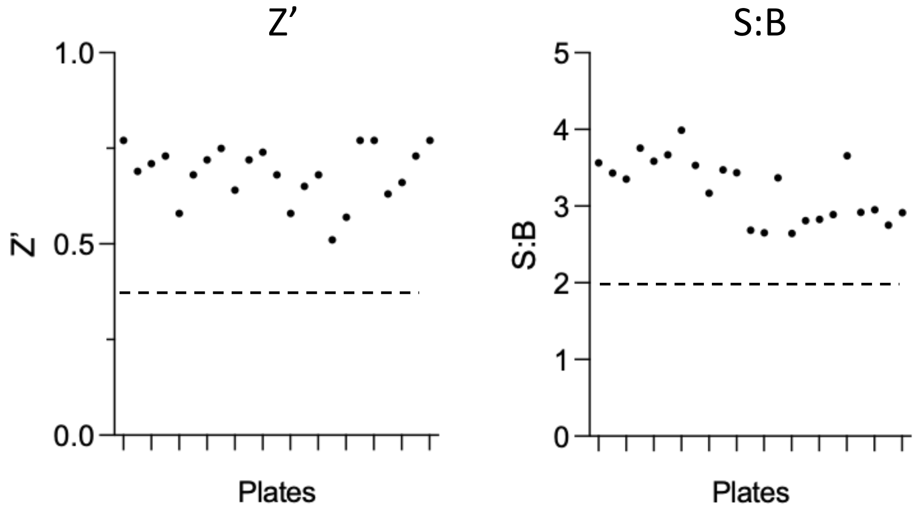


**Figure S2:** Quality control parameters used in this screening were applied to all plates. The Z' score (left panel) and the signal-to-noise ratio (S:B, right panel) were measured. The dotted line represents the threshold values: 0.4 for the Z' score and 2 for the S:B ratio.

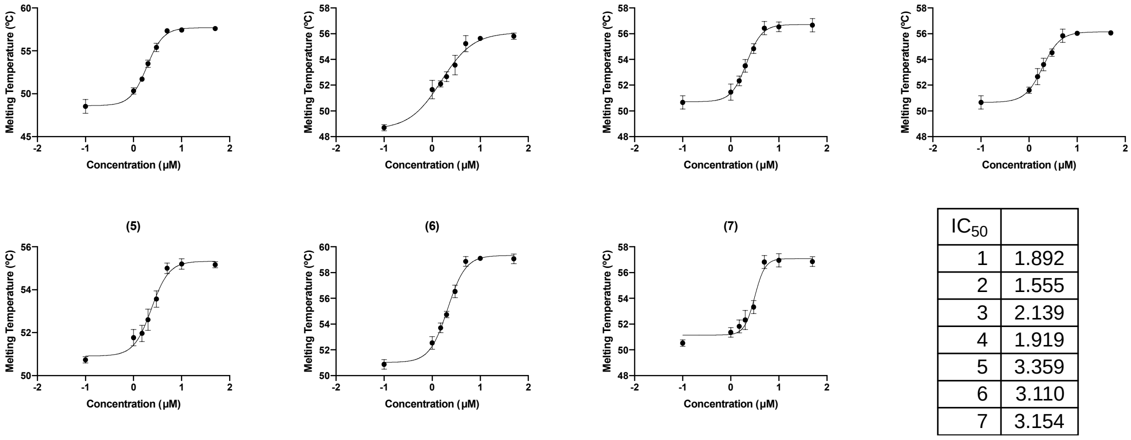


| $IC_{50}$ | |
|---|---|
| 1 | 1.892 |
| 2 | 1.555 |
| 3 | 2.139 |
| 4 | 1.919 |
| 5 | 3.359 |
| 6 | 3.110 |
| 7 | 3.154 |

**Figure S3: *Kd* calculation using DSF (second round of experiments).** Six concentrations of each compound were used (from 0.1 to 50 μM) and the melting temperatures of *Tc*BD2 with each concentration of compound obtained were used to calculate the *Kd*s. DMSO was used as a control sample. All thermal shift assays were performed at least in triplicate with reproducibility of parameters within ±10%. The change of melting temperature versus the log of the ligand concentration was plotted and fitted as described in the Methods section.

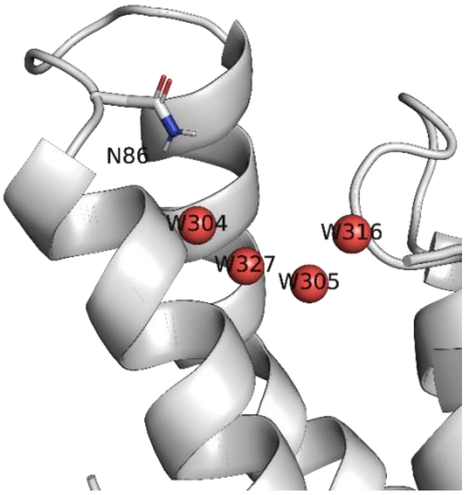


**Figure S4: Structure of *Tc*BDF2 and the water network.** Bromodomain structure with the conserved water molecules highlighted as red spheres. The four waters are referred to as W304, W305, W316, and W327.

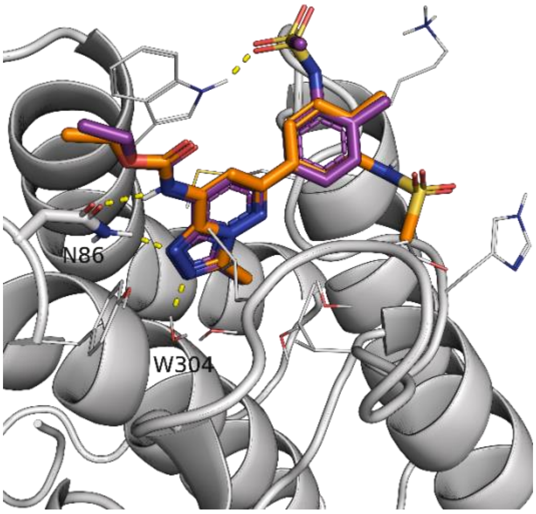


**Figure S5: Binding mode validation of the docking protocol.** Bromosporine (orange) was docked into the AcK pocket of TcBDF2 (PDBid 6NIM) obtaining an almost identical conformation as the X-ray ligand (purple) (core RMSD = 0.31 Å). Hydrogen bonds are shown in yellow dot lines.

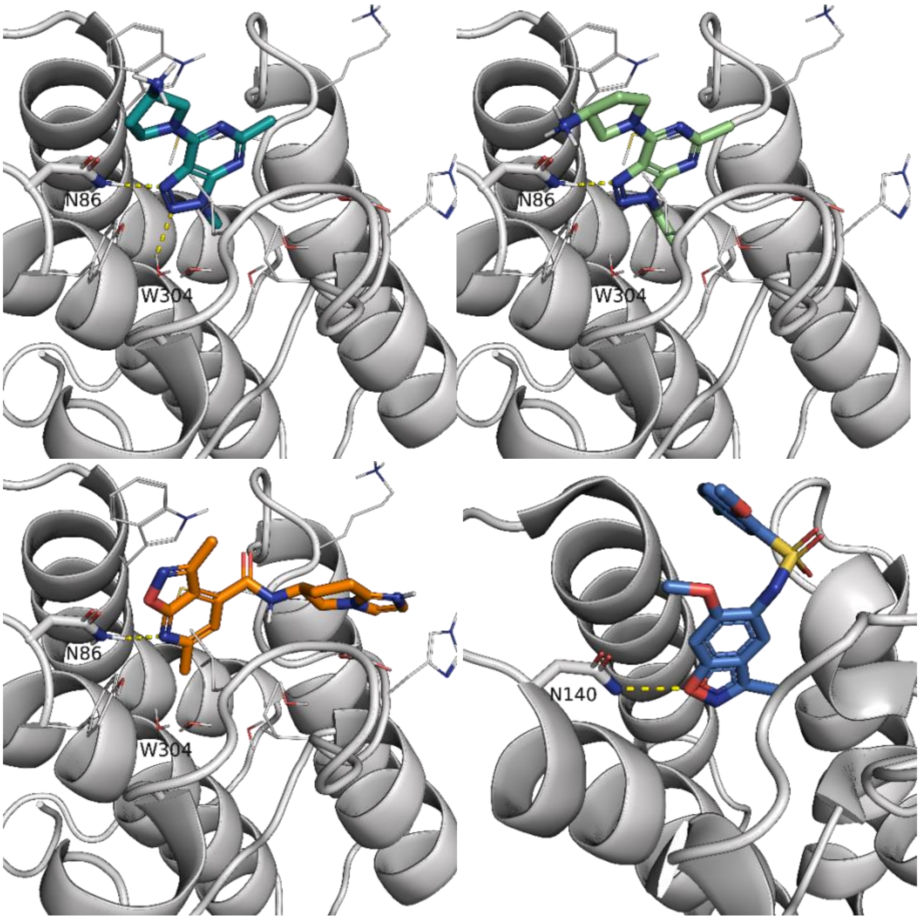


**Figure S6: Binding mode prediction of compounds against *Tc*BDF2 and X-ray crystal structure of BRD4.** Docked poses of compound 1 (dark cyan), 2 (green), 3 (orange), 3m (blue) and BRD4 (PDBid 5Y8C). Hydrogen bonds are shown in yellow dot lines.

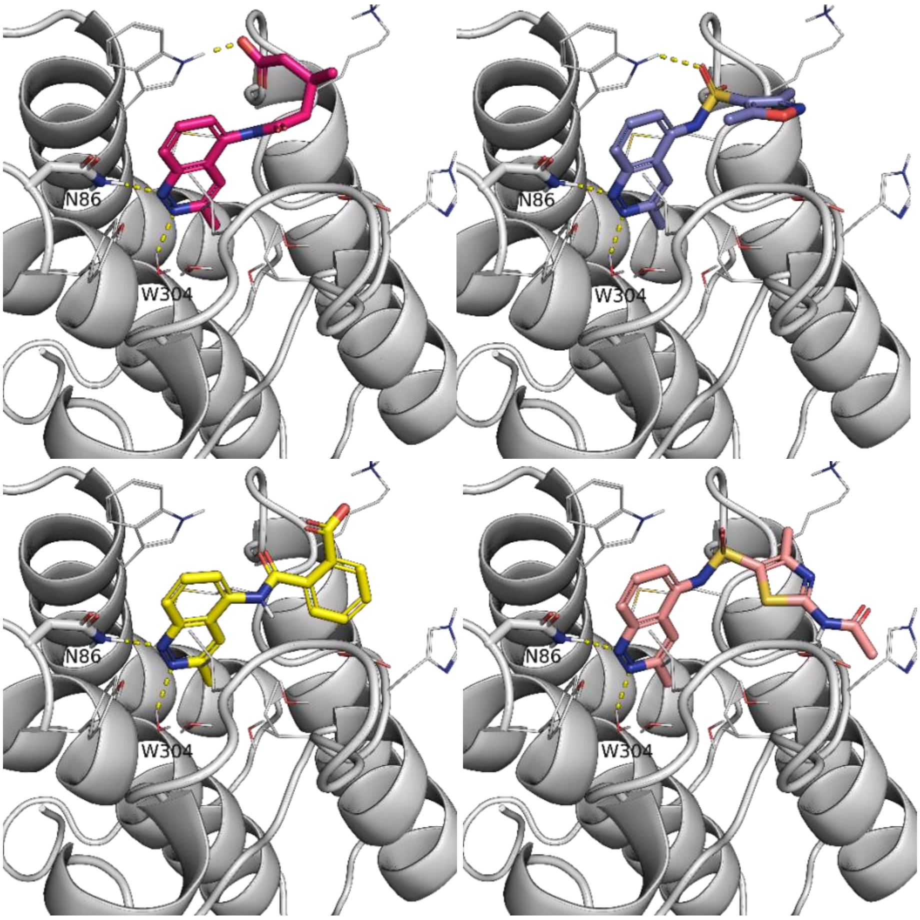


**Figure S7: Binding mode prediction of compounds against *Tc*BDF2.** Docked poses of compound 4 (dark pink), 5(purple), 6(yellow) and 7(pink). Hydrogen bonds are shown in yellow dot lines.
